# Spica Casting of Pediatric Femur Fractures: The Pain Management Experience at One Institution

**DOI:** 10.7759/cureus.28632

**Published:** 2022-08-31

**Authors:** Kevin Williams, Noor Saeed, Stephanie Ihnow, Colleen Mangeot, Jaime Denning

**Affiliations:** 1 Orthopaedic Surgery, University of Alabama at Birmingham School of Medicine, Birmingham, USA; 2 Orthopedic Surgery, Cincinnati Children's Hospital Medical Center, Cincinnati, USA; 3 Orthopedics, University of Florida College of Medicine, Gainesville, USA; 4 Biostatistics and Epidemiology, Cincinnati Children's Hospital Medical Center, Cincinnati, USA; 5 Orthopedics, Cincinnati Children's Hospital Medical Center, Cincinnati, USA

**Keywords:** pain medication, opioids, hip spica, pediatric orthopedics, femur fractures

## Abstract

Objectives

Currently, very little literature exists regarding the "fifth vital sign" in pediatric orthopedics, pain. Multiple studies have highlighted the utility of non-narcotic pain medications in treating acute pain. The objective of this study is to determine the type and amount of pain medication(s) administered and subsequently prescribed to pediatric patients ages six months to five years old with femur fractures treated with spica casting in the ER (emergency room) and OR (operative room). We also determined the incidence of spica cast change necessary for the two groups as a secondary outcome.

Methods

A retrospective review was completed at a single level 1 pediatric trauma center, evaluating 82 patients who met the inclusion criteria between six months to five years of age with isolated femoral shaft fractures requiring intervention at one institution. Descriptive statistics and Wilcoxon Rank-Sum or Fisher'sFisher's Exact test were used to assess differences between OR and ER groups for either continuous or categorical variables, respectively. The electronic medical record was then queried for demographic information, location of spica cast placement, hours in the hospital, and amount and type of analgesic medications administered and prescribed.

Results

Overall, we noted a preponderance of femur fractures in young males (72%), with the mean age of our cohort being 2.3 years old. Our patients spent a median of 20.9 hours in the hospital and had a median worst pain score of 7/10 during their hospital stay. No difference was found between standardized amounts of morphine equivalent administration between groups in the hospital. Upon discharge from the hospital, most patients received opioid and acetaminophen prescriptions (72% and 83%), but few received an ibuprofen prescription (24.4%). More spica casts placed in the ER needed to be revised in the OR compared to spica casts placed in the OR (57% vs. 8%, p<0.01).

Conclusions

There are various medication regimens for patients with femoral shaft fractures treated with spica casting at one institution. Our study revealed that patients received more prescription opioids if treated in the OR. Additionally, spica casting in the ER did not significantly decrease hospital stay, and it significantly increased the risk of needing a reduction in the OR in our institution.

## Introduction

Very little literature exists regarding the "fifth vital sign" in pediatric orthopedics, pain. Pain assessments became as important as the other four vital signs in the mid-1990s following publications from the American Pain Society and validation by Joint Commission evaluations. They standardized the approach to pain management by requiring adequate charting of pain scores both initially and after treatment [1-2]. With governing bodies paying specific attention to appropriate analgesia, orthopedic surgeons strived to augment patient satisfaction with adequate pain control. They quickly vaulted to the fourth-ranked prescribers of opioids, contributing to an "opioid epidemic" [3]. Pediatric orthopedic surgeons were not sheltered from this epidemic [4]. 

Multiple studies have shown non-narcotic pain medications, especially non-steroidal anti-inflammatories (NSAIDs) such as ibuprofen, to be equal to or better than codeine or oral morphine for controlling pain associated with fractures in children [5-9]. These studies, however, have not included femur fractures and have not included patients less than five years of age. Another study looking at pain control after outpatient orthopedic procedures showed that ibuprofen was as effective as morphine with fewer side effects [10]. This study only included minor procedures and patients five years or older.

Femur fractures comprise 1.6% of all pediatric fractures, with about one-third occurring in patients five years old or younger. Length stable femur fractures in children six months to five years of age are typically treated with a hip spica cast [11]. These are typically applied under sedation in the emergency room (ER) or the operating room (OR). After application of the cast, patients are often observed in the hospital for pain control and parental teaching. Pediatric patients with long bone fractures, including femur fractures, are often discharged on multimodal pain medication, including narcotics, acetaminophen, or anti-inflammatory medications. Currently, no guidelines or clinical standards exist to aid in appropriate post casting or discharge analgesia management for pediatric femur fractures treated with spica casting.

Our descriptive study aims to determine the type of pain medication(s) administered within the hospital and subsequently prescribed to pediatric patients ages six months to five years old with femur fractures treated with spica casting in the ER and OR.

## Materials and methods

Following review by the Institutional Review Board (IRB) granting an exemption status, a retrospective review was completed at a single level 1 pediatric trauma center. All patients (109) with isolated femoral shaft fractures requiring intervention at one institution were retrospectively identified from 2013 to 2018. Participants were included if they were between 6 months and five years of age, sustained an isolated femur fracture confirmed radiographically and underwent spica cast placement (82). Patients were excluded if they sustained multiple injuries, were outside the given age range, or were not initially treated with spica cast application. These patients were divided into two groups: 21 patients underwent cast placement in the ER, and 61 patients underwent cast placement in the OR.

The electronic medical record (EMR) was queried, and the following demographic information was collected for each patient: age, gender, mechanism of injury, associated diagnoses, and fracture characteristics. The location of spica cast placement was recorded, and the attending orthopedic surgeon typically oversaw treatment decisions. Casts were applied by various trainees (residents to fellows), usually without supervision in the ER and typically with attending physician supervision in the OR. The Cast technique was determined by the provider applying the cast and not recorded for this study. The combined orthopedic team dictated discharge planning, and one of the team members typically taught car seat teaching after spica cast application was administered prior to discharge.

We calculated the number of hours spent for each patient, from the presentation to the discharge time. The amount and type of analgesic medications were recorded throughout the patient stay. Pain scores were documented on a 0-10 scale by nursing staff with no particular standardization protocol in place. The Faces Pain Scale augmented this scale for nonverbal children to extrapolate a numerical value. Opioid administration was measured, and the measurements were standardized across patients by converting opioids administered to morphine equivalent (Meq) units and dividing by patient weight and hours in a hospital, resulting in Meq/g/hr.

Data analysis

Descriptive statistics overall and by location (ER vs. OR) were performed. For continuous variables, the median and 25th -75th percentiles were calculated. Counts and percentages were calculated for categorical variables. Wilcoxon Rank-Sum or Fisher'sFisher's Exact test was used to assess differences between OR and ER groups for either continuous or categorical variables, respectively. Alpha of 0.05 was used to determine statistical significance.

## Results

Of the 109 patients identified with isolated femoral shaft fractures, 27 were excluded based on the above criteria, making 82 patients available for analysis. Demographic and descriptive statistics with statistical test results between the two groups are shown in Table 1.

**Table 1:** Demographic and descriptive statistics with statistical test results between OR and ER groups.

	Overall (n=82)	ER (n=21)	OR (n=61)	p-value
Average Age in Years	2.3	2.5	2.3	0.303
Gender				
Female	23 (28.0%)	7 (33.3%)	16 (26.2%)	0.579
Male	59 (72.0%)	14 (66.7%)	45 (73.8%)	
Mechanism of Injury				
Fall	55 (67.1%)	15 (71.4%)	40 (65.6%)	1.000
Non-accidental Trauma (NAT)	6 (7.3%)	1 (4.8%)	5 (8.2%)	
Other	21 (25.6%)	5 (23.8%)	16 (26.2%)	
Associated Diagnosis Present				
No	71 (86.6%)	19 (90.5%)	52 (85.2%)	0.720
Yes	11 (13.4%)	2 (9.5%)	9 (14.8%)	
Fracture Location				
Distal	10 (12.2%)	5 (23.8%)	5 (8.2%)	0.162
Mid Shaft	52 (63.4%)	11 (52.4%)	41 (67.2%)	
Proximal	20 (24.4%)	5 (23.8%)	15 (24.6%)	
Fracture Type				
Spiral	60 (73.2%)	16 (76.2%)	44 (72.1%)	0.783
Transverse	22 (26.8%)	5 (23.8%)	17 (27.9%)	
Reduction in OR				
No	65 (79.3%)	9 (42.9%)	56 (91.8%)	<0.001
Yes	17 (20.7%)	12 (57.1%)	5 (8.2%)	
Worst Pain Score Recorded(x/10)*	7.0	7.0	7.0	0.696
Hours in Hospital	20.9	19.9	21.6	0.188
Morphine Equivalents Given in Hospital	9.8	8.1	10.2	0.836
Morphine RX				
No	23 (28.0%)	10 (47.6%)	13 (21.3%)	0.027
Yes	59 (72.0%)	11 (52.4%)	48 (78.7%)	
Tylenol RX				
No	14 (17.1%)	6 (28.6%)	8 (13.1%)	0.175
Yes	68 (82.9%)	15 (71.4%)	53 (86.9%)	
Ibuprofen RX				
No	62 (75.6%)	17 (81.0%)	45 (73.8%)	0.572
Yes	20 (24.4%)	4 (19.0%)	16 (26.2%)	

Overall, we noted a preponderance of femur fractures in young males (72%), and the median age was 2.3 years. Most injuries resulted from a fall (67%), and the fewest from non-accidental trauma NAT (7%). Eleven patients (13%) had a diagnosis potentially predisposing them to a femur fracture. Most fractures followed a spiral fracture pattern in the midshaft of the femur, though there was some variety in our particular patient population in location and type. Our patients spent a median of 20.9 hours in the hospital and had a median worst pain score of 7/10 during their hospital stay. 

Medication administration showed a wide variety of opioid administration patterns. Patients received between 0 and 58.3 Meq/g/hr. The median, 25th, and 75th percentiles were 9.8, 5.1, and 16.9, respectively. Upon discharge from the hospital, most patients received opioid and acetaminophen prescriptions (72% and 83%), but few received an ibuprofen prescription (24.4%).

The OR and ER groups were similar in demographic data and associated diagnoses. A more significant percentage of distal fractures were treated in the ER, but it was not statistically significant. Significantly more spica casts in the ER returned to the OR than spica casts in the OR (57% vs. 8%, p<0.01). The OR group trended toward spending more time in the hospital at 21.6 hours, whereas the ER group averaged 19.9 hours (P = 0.19).

**Figure 1:**
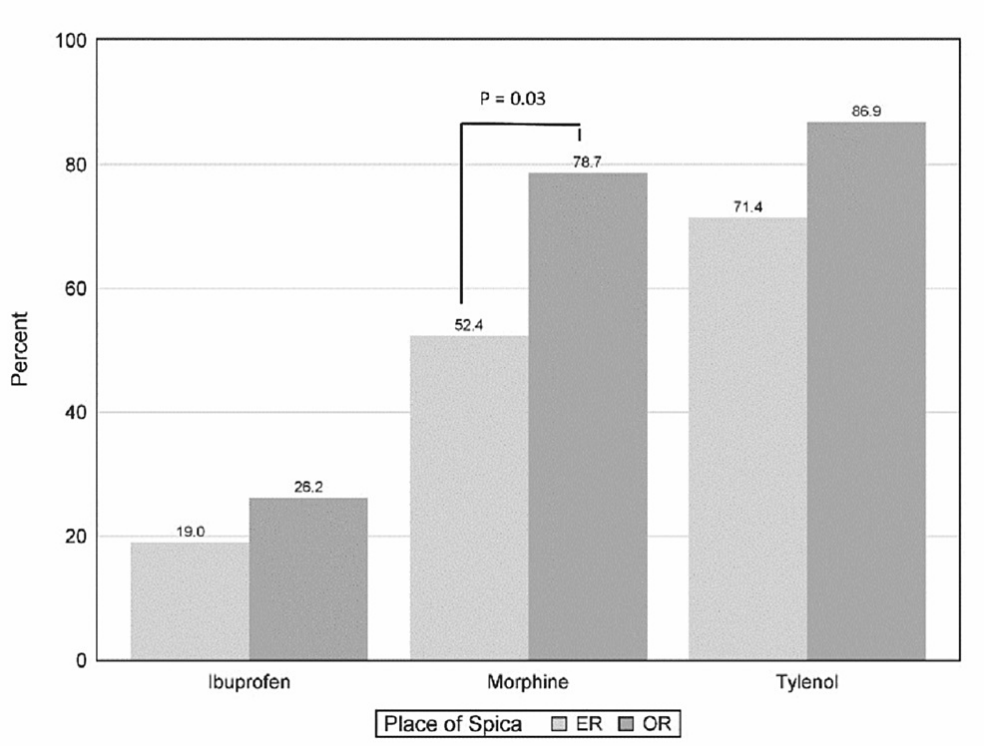
Discharge Analgesia Prescriptions Between ER and OR Groups

No difference was found between standardized amounts of morphine equivalent administration between groups in the hospital. There was, however, a significant difference between opioids prescribed at discharge between groups, with a more significant percentage of patients in the OR group prescribed opioid-type medications (79% vs. 52%, p=0.03). They also trended toward receiving more acetaminophen-type prescriptions (p=0.17). Overall, very few patients in both groups were prescribed ibuprofen. Figure 1 represents the percentage of patients prescribed different types of medication for the two groups.

## Discussion

The national opioid crisis is a severe health crisis that affects all levels of patient care, including the individual, family unit, public health sphere, and social and economic welfare. One hundred twenty-eight Americans die daily from opioid overdose, and the CDC estimates the economic burden of prescription opioid use as $78.5 billion per year. Therefore, over the past few decades, the U.S. Department of Health and Human Services and the National Institute of Health have shifted focus on decreasing opioid prescriptions for pain [12]. Despite these efforts to reduce ongoing opioid use, little literature on the pediatric population currently exists. Many children are first exposed to opioids following injury and its subsequent treatment. We thus chose to study pediatric femur fractures treated with spica casting and its subsequent pain management regimen to elucidate these patients' pain medication needs and to help develop a basis for guidelines and clinical standards for pain control in the future.

In the U.S., femur fractures are the third most common lower extremity fracture and the most common orthopedic injury requiring pediatric hospitalization. Despite formal guidelines regarding femur fracture treatment, there is no consensus on the best treatment due to age, fracture pattern, mechanism, and associated injury [13]. Current treatment options include spica casting, Pavlik harness application, intramedullary nailing, plating, and external fixation [14, 15]. A 2018 review found that spica casting was the most preferred treatment among North American pediatric orthopedists for patients <6 years old. They identified several characteristics associated with the failure of spica cast treatment, including excessive angulation and shortening, based on age [16]. A 2010 study compared children 1 to 4 years of age with femoral shaft fractures treated by spica casting versus flexible intramedullary nailing. The spica group underwent less general anesthesia throughout their treatment protocols, but overall, both groups had similar outcomes and complication rates [17].

The demographic makeup of our patient group fits that most commonly described in these studies: a preponderance of male gender of walking age. Most injuries resulted from a fall, which is also consistent with described mechanisms mentioned above. Eleven out of 82 patients had an associated diagnosis that could increase their risk for a femur fracture. We initially anticipated that these would be associated with spica placement in the OR; however, we determined this was not necessarily the case as they were not statistically related.

Although the majority of femur fractures are isolated injuries due to trauma, they can also be associated with polytrauma and child abuse, especially in infants. A meta-analysis by Wood et al. found a substantial risk of non-accidental trauma (16.7% - 35.5%) in infants < 12mo old with a femur fracture. This risk decreased substantially (1.5% - 6.0%) once the child reached walking age [18]. Our particular cohort traverses both age groups. Our rate of femur fractures associated with NAT seems consistent with the literature, at 7.3% of our cohort.

New literature has recently emerged regarding opioids for orthopedic surgery, though the amount of pediatric-related literature is scant. Chen et al. released evidence that patient-specific opioid tapers can decrease the number of postoperative opioids administered following several different opioid procedures without untoward perceptions by the patients [19]. Additionally, in the pediatric literature, a prospective study was performed to quantify the amount of analgesia taken by patients 12-18 years of age undergoing anterior cruciate ligament reconstruction. With their standardized opioid administration protocol, the median number of opioids taken per patient was 17 (range from 0 to 40), with no difference noted amongst patient or surgical variables [20].

Our study did not determine the amount taken after hospitalization but the amount prescribed following discharge. Interestingly, we found a significant difference in opioid prescriptions between groups. This may have resulted from different providers administering different prescriptions, given no protocol. Acetaminophen was prescribed 83% of the time, but ibuprofen was only prescribed 24% of the time. These may be somewhat confounded due to providers simply instructing patients to take over-the-counter medications. We were surprised at the lack of ibuprofen prescriptions nonetheless, given the safety and efficacy of the medication to decrease opioid intake demonstrated by multiple randomized trials [21]. Pain scores were similar across our patient groups; therefore, the discharge prescriptions were expected to be similar between the two groups.

There was no difference between groups in terms of morphine administered in the hospital based on our standardized morphine equivalency calculations. There was subsequently no difference in the number of hours in the hospital between groups. The median number of hours in the hospital for our cohort was 20.9. Performing spica casting in the ER did not correlate with a significantly decreased hospital stay but was significantly correlated with a return to the OR. This could be a function of trainees (fellows and residents) applying the cast or the lack of adequate supplies, fluoroscopy, academic help, or appropriate sedation within the emergency department setting.

Additionally, we did not analyze the indications for returning to the OR, which could have differed among providers. Our current protocol does include the application of casting in the ER. Mansour et al. provided a retrospective review that demonstrated comparable complication rates between OR and ER spica cast application; however, ER application resulted in less time for spica placement, fewer hours in the hospital, and decreased hospital charges [22]. We did not record an overall complication rate; however, our results differed in overall return visits to the OR. Our ER cohort had a 57% rate of return to the OR as opposed to our OR cohort, which was 8%, and more in line with the previously reported values.

Limitations to our study include those associated with a retrospective review of the medical record. It is somewhat tricky overall to ascertain the validity of the recorded information, especially related to the timing and accuracy of medication administration and prescription provision. Additionally, there is no standardized protocol within our institution involving analgesia administration, and some patients even undergo evaluation by a pain service, which could confound the study. Further, providers will occasionally recommend over-the-counter analgesia therapy without providing a prescription, which could confuse the overall prescription counts. Also, it is essential to note that prescription administration does not correlate with medications taken at home, which we did not study. We did not record many aspects of patient care for this study, including patient or radiographic outcomes, pain scores at home, or the indications for returning to the OR.

## Conclusions

Our study demonstrated that pain medication administration during hospitalization and provision of discharge prescriptions varied widely in a retrospective study performed at one institution. This discovery warrants additional prospective studies regarding pain medication administration and further non-opioid alternatives to add to an institution's arsenal for managing pain associated with musculoskeletal pathologies. Despite literature revealing NSAID efficacy and a good safety profile, this has not translated to increased utilization of NSAIDs either during hospitalization or upon discharge at our institution. Our goal is to develop literature-based pain management guidelines and implement them prospectively. This will allow us to add to the literature by comparing inpatient and outpatient pain management in a similar patient population undergoing hip spica casting for pediatric femur fractures and other musculoskeletal pathologies.
